# Impact of the Coronavirus Disease 2019 (COVID-19) Curfew Restrictions on the Body Mass Index and Dietary and Physical Activity Behaviors of Saudi Adults: A Cross-Sectional Study

**DOI:** 10.7759/cureus.52669

**Published:** 2024-01-21

**Authors:** Suzan H Tami, Sabiha Khan, Rafia Bano

**Affiliations:** 1 Department of Food and Nutrition Sciences, King Faisal University, Hofuf, SAU; 2 School of Public Health and Primary Care, Fiji National University, Suva, FJI; 3 Department of Clinical Nutrition, King Faisal University, Hofuf, SAU

**Keywords:** body mass index, pandemic, covid-19, physical activity, diet

## Abstract

Objectives: The objective of the current study was to assess the effect of the coronavirus disease 2019 (COVID-19) curfew restrictions on the body mass index (BMI) and dietary and physical activity behaviors of Saudi adults.

Methods: This cross-sectional study was conducted using an online questionnaire in Saudi Arabia in May and June 2020, during the curfew restriction period. The questionnaire included three sections with questions regarding sociodemographic data, dietary behaviors, and moderate and vigorous activities.

Results: A total of 504 Saudi adults participated in this study. Even though there were no significant differences in the BMI status before and during COVID-19 curfew restrictions, the percentage of overweight participants slightly increased during the period of COVID-19 curfew restrictions. The frequency of intake of fruits and beverages were significantly increased (p<0.05), whereas the consumption of meat and fish, bread and cereals, and sandwiches and burgers showed a significant reduction (p=0.001). Although the overall percentage of participants engaged in moderate physical activities increased during curfew restrictions, there was a reduction in vigorous physical activities.

Conclusion: This study demonstrated changes in BMI and dietary and physical activity behaviors due to the COVID-19 curfew restrictions.

## Introduction

The coronavirus disease 2019 (COVID-19) is an infectious disease and may cause mild to moderate respiratory illnesses such as the Middle East respiratory syndrome (MERS-CoV) and severe acute respiratory syndrome (SARS-CoV) [1,2]. However, elderly people and those with underlying medical conditions (such as cardiovascular disease, diabetes, chronic respiratory disease, or cancer) are more likely to develop serious illnesses. Up to now, there have been over 750,000,000 confirmed cases of COVID-19, including nearly 7,000,000 deaths, reported to WHO [3]. The rapid increase of COVID-19 cases has challenged the capacity of healthcare systems.

Once COVID-19 was declared a pandemic in March 2020 [2], most countries started drastic strategies like travel bans, lockdowns, and curfew restrictions to prevent COVID-19 spread during its peak. That, in turn, led to challenges in lifestyles, including dietary and physical activity behaviors, among people across the globe [4-6]. Specifically, drastic strategies of curfew restrictions and closures of restaurants might impact individuals' dietary behaviors through increased "comfort food" consumption due to emotional distress. In addition, curfew restrictions and lockdowns of parks and fitness centers might disrupt physical exercise routines, limit outdoor activities, and encourage sedentary behaviors.

The Saudi Ministry of Health (MOH) announced the first case of COVID-19 infection on March 2, 2020 [1]. In April 2020, the Saudi government announced a curfew for 24 hours a day in cities that witnessed a high number of COVID-19 cases and excluded leaving the house for necessary needs through an application that granted electronic permits to employees of government and private sectors [1]. Soon after, all Saudi cities introduced similar measures, mainly enforcing social distancing by the closure of restaurants, sport centers, and cultural facilities as well as prohibiting mass gatherings and interaction of people from different households. Most Saudi individuals had to experience online working, learning, and attending/conducting meetings for the first time.

Being forced to stay home due to the COVID-19 pandemic for a long time might eventually lead to stress conditions that could cause a dramatic change in individuals' dietary and lifestyle behaviors [7]. A study by Pietrobelli et al. [8] found increased consumption of unhealthy foods and decreased physical activities among Italians during the lockdown. In addition, Janssen and others used a food frequency questionnaire and found an overall reduction in the consumption of fresh foods and an increased intake of foods with a longer shelf life in Denmark and Germany [9]. Decreased consumption of fresh foods (especially fruit and fish) and increased consumption of sweets, cookies, and cakes were also reported in France as well as decreased physical activity and increased sedentary time [10]. Such dietary and physical activity behaviors might have impacted individuals' weight as well [11,12].

While much of the emerging research investigated weight changes, eating habits, and physical activities of different populations due to COVID-19 curfew shutdowns [13,14], a few studies have focused on changes in both dietary and physical activity behaviors of Saudi populations during the COVID-19 pandemic [14-18]. Moreover, no previous research, to our knowledge, reported changes related to dietary behaviors using a validated Saudi food frequency questionnaire (SFFQ) nor measured changes in both moderate and vigorous physical activity behaviors in Saudi adults during the COVID-19 curfew restrictions. The current study aimed to assess the effect of COVID-19 curfew restrictions on body mass index (BMI) and dietary and physical activity behaviors of Saudi adults.

## Materials and methods

This cross-sectional study was conducted at the end of May and the month of June 2020 (during the COVID-19 curfew restrictions) using convenience and snowball sampling methods of both Saudi female and male adults (18 and above). The data was collected through the Google Forms platform, and the online survey (see Appendices) was distributed via social media networks (Twitter, Snapchat, and WhatsApp). The study protocol and questionnaire were approved by the Research Ethics Committee of King Faisal University (approval number: KFU-REC/2020-05-14).

The first page of the survey included the objectives of the study, the duration of the survey, voluntary declaration, confidentiality, the anonymity of the study, and a digital consent form. The study participants were required to provide consent of participation before they started the survey by clicking on the "agree to participate in this study" button. The participants also had the option to exit the survey at any point if they felt uncomfortable or did not want to continue participating in the study.

The study survey was available in Arabic for the participants and consisted of three sections. The first section collected sociodemographic data (including gender, age, marital status, education level, occupation, region of residence, and monthly household income level). A closed-ended question on a change of household income during the curfew restrictions was provided with options (no; yes: increased income; and yes: decreased income). The survey also included questions on height and body weight (before and during the curfew restrictions) to calculate BMI with the formula of weight in kilograms divided by height in meters squared and categorize it as underweight (below 18.5), normal weight (18.5-24.9), overweight (25-29.9), and obese (30 and above) [19].

The second section of the survey collected dietary behaviors before and during the curfew restrictions using an SFFQ. The SFFQ was developed by Gosadi et al., and its reliability and validity were established [20]. Each set (before and during the curfew restrictions) of the SFFQ included a list of 140 food items of eight food categories (meat and fish, bread and cereals, sandwiches and burgers, dairy products and fat, sweets and snacks, beverages, fruits, and vegetables), and nine answer options were provided for each closed-ended question. The consumption frequency choices were stated as the following: never or less than a month, 1-3 per month, once a week, 2-4 per week, 5-6 per week, once a day, 2-3 per day, 4-5 per day, and 6+ per day. At the end of the food frequency questionnaire, open-ended questions were provided to gather information regarding other non-listed food items.

The third section included questions on both moderate and vigorous physical activities before and during the curfew restrictions. Definitions of both moderate and vigorous physical activities [21] were provided to the study participants with the following statements: "moderate physical activity refers to activities equivalent in intensity to brisk walking, bicycling, cleaning the house, or landscaping the garden" and "vigorous physical activity produces large increases in breathing or heart rate, such as jogging, fitness exercises, or hard-working in the house garden." The physical activity questions were based on the Behavioral Risk Factor Surveillance System (BRFSS) Questionnaire [22]. For each type of physical activity, participants were asked about the number of days and the total amount of time (minutes/week) by open-ended questions.

Statistical analysis was performed using IBM SPSS Statistics for Windows, Version 27.0 (Released 2020; IBM Corp., Armonk, New York, United States). Categorical data were presented in frequencies and percentages, and continuous data were expressed in means and standard deviations. The normal distribution of a variable was evaluated using the Kolmogorov-Smirnov test. To measure dietary behaviors, a scoring system was used. For example, food items which were never or rarely consumed were given a score of 0, while food items which were consumed more than six times daily were given a score of 8. A sum of consumption frequency score was calculated for each food item and the eight food categories. The paired sample t-test or Wilcoxon signed-rank test was used to compare the total scores for food frequency consumption and physical activity behaviors before and during COVID-19 curfew restrictions. The McNemar-Bowker test was used to compare the BMI status before and during COVID-19 curfew restrictions. A p-value of less than 0.5 was considered statistically significant. To measure the food consumption frequency, a scoring system was used. For example, items which were never or rarely consumed were given a score of 0, while items which were consumed more than six times daily were given a score of 8. A sum of consumption frequency score was calculated for each food type.

## Results

In this study, 537 individuals completed the online survey. After eliminating non-Saudi participants (n=19) and duplicated entries (n=14), the participation sample included 504 Saudi adults (18 years old and above). The study participants' characteristics are presented in Table 1. The majority of the study participants were female (n=379, 75.2%), had a bachelor's degree (n=289, 57.3%), were employed (n=207, 41.1%), and were married (n=244, 48.4%). The age mean of the participants was 31.6(±10.21). About 50% (n=253) of the study participants were from the eastern region. Over 60% (n=326) of the participants had a monthly household income of SR 10,000 (~USD 2,666) or above, and their income did not change during the COVID-19 curfew.

**Table 1:** Demographic characteristics of the study participants (n=504)

Item	n	%	M(±SD)
Gender			
Female	379	75.2	
Male	125	24.8	
Age			31.6(±10.21)
Education level			
Less than high school	11	2.2	
High school or equivalent	68	13.5	
Some college	51	10.1	
Bachelor's degree	289	57.3	
Post-graduate degree	85	16.9	
Occupation			
Student	142	28.2	
Employed	207	41.1	
Unemployed	155	30.8	
Marital status			
Married	244	48.4	
Single	232	46	
Divorced and widowed	28	5.6	
Region of residence			
Eastern region	253	50.2	
Western region	113	22.4	
Central region	83	16.5	
Southern region	43	8.5	
Northern region	12	2.4	
Monthly household income			
Over SR 19,000	117	23.2	
SR 14,000-19,000	103	20.4	
SR 10,000-13,999	106	21	
SR 6,000-9,999	87	17.3	
Under SR 6,000	91	18.1	
Change in income during COVID-19 curfew			
No	321	63.7	
Yes: decreased income	142	28.2	
Yes: increased income	41	8.1	

Table 2 presents the study participants' BMI before and during COVID-19 curfew restrictions. Based on the self-reported weight and height before and during the COVID-19 curfew restrictions, about 8% (n=40) of the participants were underweight before COVID-19 curfew restrictions, and about 7% (n=35) of the participants were underweight during COVID-19 curfew restrictions. Out of the study participant, 40.7% (n=205) had a normal weight before COVID-19 curfew restrictions, and 42.1% (n=212) of the participants had a normal weight during COVID-19 curfew restrictions. In addition, 28.6% (n=144) of the participants were overweight before COVID-19 curfew restrictions, and almost 30% (n=149) of the participants were overweight during COVID-19 curfew restrictions. Furthermore, 22.8% (n=115) of the participants were obese before COVID-19 curfew restrictions, and 21.4% (n=108) of the participants were obese during COVID-19 curfew restrictions. However, there were no significant differences in the BMI status before and during COVID-19 curfew restrictions.

**Table 2:** Study participants' BMI before and during COVID-19 curfew restrictions (n=504) BMI: body mass index; COVID-19: coronavirus 2019

BMI category	Before COVID-19 curfew (%)	During COVID-19 curfew (%)	p-value
			0.087
Underweight (below 18.5)	40 (7.9)	35 (6.9)	
Normal weight (18.5-24.9)	205 (40.7)	212 (42.1)	
Overweight (25-29.9)	144 (28.6)	149 (29.6)	
Obese (30 and above)	115 (22.8)	108 (21.4)	

Table 3 shows the study participants' dietary behaviors before and during the COVID-19 curfew restrictions. It was found that the consumption of meat and fish, bread and cereals, and sandwiches and burgers was significantly (p=0.001) reduced during the COVID-19 curfew period. On the other hand, the intake of sweets and snacks (p=0.015), beverages (p=0.001), and fruits (p=0.010) showed a significant increase. The change in consumption of dairy products and fat and vegetables before and during the COVID-19 curfew restrictions was found to be statistically insignificant.

**Table 3:** Study participants' dietary behaviors before and during the COVID-19 curfew restrictions (n=504) *p-value is considered significant at <0.05 COVID-19: coronavirus 2019

Food category	Before COVID-19 curfew	During COVID-19 curfew	Diff (B-A)	p-value
Meat and fish	16.63±9.8	14.98±9.7	1.64 (1.22, 2.06)	0.001*
Bread and cereals	22.92±12.34	21.99±12.21	0.93 (0.47, 1.39)	0.001*
Sandwiches and burgers	11.76±7.74	8.94±7.04	2.82 (2.47, 3.17)	0.001*
Dairy products and fat	23.21±16.58	23.54±16.93	-0.33 (-0.73, 0.071)	0.075
Sweets and snacks	25.35±16.8	26.12±17.41	-0.778 (-1.51, -0.049)	0.015*
Beverages	13.62±9.65	14.09±9.99	-0.466 (-0.818, -0.114)	0.001*
Fruits	21.63±17.13	22.16±17.01	-0.538 (-1.11, 0.034)	0.010*
Vegetables	34.47±22.63	34.87±21.97	-0.395 (-0.976, 0.186)	0.182

Table 4 shows the results of the univariate regression analysis of BMI depending on consumption frequency within each food category of study participants before and during COVID-19 curfew restrictions. It was concluded that the results were statistically not significant at p<0.05.

**Table 4:** Univariate regression analysis of BMI depending on consumption frequency within each food category of study participants before and during COVID-19 curfew restrictions (n=504) BMI: body mass index; COVID-19: coronavirus 2019

Food category	Before COVID-19 curfew			During COVID-19 curfew		
	Regression coefficient	95% confidence intervals	p-value	Regression coefficient	95% confidence intervals	p-value
Meat and fish	-0.018	-0.17 to 0.13	0.810	-0.064	-0.21 to 0.09	0.402
Bread and cereals	-0.066	-0.19 to 0.054	0.279	-0.11	-0.23 to 0.009	0.071
Sandwiches and burgers	0.177	-0.035 to 0.390	0.102	-0.052	-0.16 to 0.26	0.629
Dairy products and fat	0.008	-0.081 to 0.097	0.861	0.015	-0.07 to 0.102	0.729
Sweets and snacks	-0.035	-0.123 to 0.053	0.431	-0.032	-0.11 to 0.048	0.428
Beverages	0.086	-0.068 to 0.239	0.273	0.87	-0.06 to 0.23	0.247
Fruits	0.024	-0.062 to 0.110	0.586	-0.011	-0.09 to 0.075	0.797
Vegetables	0.052	-0.013 to 0.118	0.115	0.044	-0.023 to 0.111	0.199

As for practicing physical activity, 83.2% (n=420) of the participants reported practicing at least 10 minutes of moderate physical activity per week (data not shown). In addition, 55.6% (n=281) of the participants reported practicing at least 10 minutes of vigorous physical activity per week. Table 5 illustrates study participants' physical activity behaviors before and during COVID-19 curfew restrictions. As for practicing moderate physical activity, the number of participants reporting zero days of practicing weekly moderate physical activity before COVID-19 curfew restrictions reduced from 12.5% (n=63) to 8.5% (n=43) during COVID-19 curfew restrictions, whereas the number of participants who reported 1-3 days of practicing weekly moderate physical activity before COVID-19 curfew restrictions increased from 39% (n=195) to 42.9% (n=216) during COVID-19 curfew restrictions. In addition, the number of participants reporting 4-6 days of practicing weekly moderate physical activity before COVID-19 curfew restrictions were almost the same 48.8% (n=246), as compared to 48.6% (n=246) during COVID-19 curfew restrictions. It was concluded that the numbers of days of practicing weekly moderate physical activity were statistically significant (p=0.012).

**Table 5:** Study participants' physical activity behaviors before and during COVID-19 curfew restrictions (n=504) *p-value is considered significant at <0.05 COVID-19: coronavirus disease 2019

Item	Before COVID-19 curfew (%)	During COVID-19 curfew (%)	p-value
Number of days of practicing weekly moderate physical activity			0.012*
0	63 (12.5)	43 (8.5)	
1-3	195 (38.7)	216 (42.9)	
4-6	246 (48.8)	245 (48.6)	
Number of minutes of practicing daily moderate physical activity			0.799
0-30	285 (56.5)	290 (57.5)	
31-60	155 (30.8)	137 (27.2)	
61-90	17 (3.4)	21 (4.2)	
91-120	29 (5.8)	38 (7.5)	
Over 120	18 (3.6)	17 (3.6)	
Number of days of practicing weekly vigorous physical activity			0.436
0	117 (23.2)	117 (23.2)	
1-3	246 (48.8)	248 (49.2)	
4-6	141 (28)	139 (27.6)	
Number of minutes of practicing daily vigorous physical activity			0.832
0-30	355 (70.4)	363 (72)	
31-60	102 (20.2)	94 (18.7)	
61-90	15 (3)	18 (3.6)	
91-120	25 (5)	20 (4)	
Over 120	7 (1.4)	9 (1.8)	

The total number of participants reporting 0-30 minutes of practicing daily moderate physical activity before COVID-19 curfew restrictions reduced from 65.5% (n=285) to 57.5% (n=290) during COVID-19 curfew restrictions. Also, the number of participants who reported 31-60 minutes of practicing daily moderate physical activity before COVID-19 curfew restrictions decreased from 30.8% (n=155) to 27.2% (n=137) during COVID-19 curfew restrictions. On the other hand, the percentage of the participants who reported 61-90 minutes of practicing daily moderate physical activity before COVID-19 curfew restrictions increased from 3.4% (n=17) to 4.2% (n=21) during COVID-19 curfew restrictions. Furthermore, the number of participants who reported 91-120 minutes of practicing daily moderate physical activity before COVID-19 curfew restrictions rose from 5.8% (n=29) to 7.5% (n=38) during COVID-19 curfew restrictions. Moreover, the number of participants reporting over 120 minutes of practicing daily moderate physical activity before COVID-19 curfew restrictions remained the same at 3.6% (n=18), as compared to 3.6% (n=17) during COVID-19 curfew restrictions. However, it was concluded that the numbers of minutes of practicing daily moderate physical activity were statistically not significant at p<0.05.

As for practicing vigorous physical activity, 23.2% (n=117) of the participants reported zero days of practicing weekly vigorous physical activity before and during COVID-19 curfew restrictions. Also, the number of participants who reported 1-3 days of practicing weekly vigorous physical activity before COVID-19 curfew restrictions was the same at 49% (n=246) as compared to 49.2% (n=248) of the participants during COVID-19 curfew restrictions. In addition, the percentage of the participants reporting 4-6 days of practicing weekly vigorous physical activity before COVID-19 curfew restrictions reduced from 28% (n=141) to 27.6% (n=139) during COVID-19 curfew restrictions. Nevertheless, it was concluded that the numbers of days of practicing weekly vigorous physical activity were statistically not significant at p<0.05.

Over 70% (n=355) of the participants reported 0-30 minutes of practicing daily vigorous physical activity before COVID-19 curfew restrictions, as compared to 72% (n=363) during COVID-19 curfew restrictions. Also, the number of participants reporting 31-60 minutes of practicing daily vigorous physical activity before COVID-19 curfew restrictions reduced from 20.2% (n=102) to 18.7% (n=94). In addition, only 3% (n=15) of the participants reported 61-90 minutes of practicing daily vigorous physical activity before COVID-19 curfew restrictions, as compared to 3.6% (n= 18) during COVID-19 curfew restrictions. Furthermore, there was a reduction in the number of participants who reported 91-120 minutes of practicing daily vigorous physical activity before COVID-19 curfew restrictions from 5% (n= 25) to 4% (n=20). Moreover, 1.4% (n=7) of the participants reported over 120 minutes of practicing daily vigorous physical activity before COVID-19 curfew restrictions as against 1.8% (n=9) during COVID-19 curfew restrictions. Yet, it was concluded that the numbers of minutes of practicing weekly vigorous physical activity were statistically not significant at p<0.05.

## Discussion

As a result of the COVID-19 pandemic, different countries had forced strict measures to manage the spread of the disease. That, in turn, could directly or indirectly affect eating and physical activity behavior. Like other countries, Saudi Arabia experienced diverse magnitudes of social distancing actions, which included the shutting down of sports events and places and limiting the number of customers as well as the total timing of the restaurant's opening hours. Even though these strict measures might decrease the virus communication in many parts of the world including Saudi Arabia [14-16], these procedures had augmented worries about following a healthy lifestyle including eating patterns and exercise behaviors. The current study assessed the effect of COVID-19 curfew restrictions on the BMI and dietary and physical activity behaviors of 504 Saudi adults.

In terms of BMI change, Hossain et al. reported increased BMI within prolonged (over a year) COVID-19 lockdown in Bangladeshi university students [4]. However, there were no significant differences in the BMI status before and during COVID-19 curfew restrictions in Saudi adults who participated in the current study. The study was done in a relatively short period of time; BMI change would occur overtime. Another important indicator of health is dietary behavior. It has been concluded that a poor dietary regimen would negatively affect mental well-being and cognitive performance [8,13]. The present study showed a significantly increased intake of beverages, fruits, and sweets and snacks. This observation was reported in other populations too. Specifically, a higher intake of fruits was depicted, whereas no change was found in vegetable consumption in Italian populations during the COVID-19 lockdown [8,23]. In addition, a higher intake of sweets, cookies, cakes, and sugar-sweetened beverages was found in other populations due to the COVID-19 lockdown [10,24]. It should also be considered that during the pandemic curfew, there was limited time for the public to do shopping for meal preparation. Moreover, people could feel pressured concerning the food reserves for the future, and that might lead them to getting pre-packaged and non-perishable eatables and "comfort foods" such as sweets and snacks in place of fresh ones. That, in turn, might lead to the consumption of excessive amounts of calories and overweight [25]. To avoid negative consequences, the efforts of public health officials should include increased awareness on healthy eating during pandemics.

Being physically active is the most powerful determinant of decreased anxiety and depression [26], and it also contributes to weight control and develops healthy immunity. While previous studies suggested reduced physical activities during the pandemic lockdowns [10,17, 27-29], the number of days the Saudi participants reported practicing moderate physical activities significantly increased (p=0.012) as a result of the COVID-19 curfew restrictions. One explanation to increased moderate physical activities in Saudi adults during the COVID-19 curfew restrictions might be having extra time while staying home. However, no significant change was found regarding practicing vigorous physical activities because of the COVID-19 curfew restrictions. The study took place during the pandemic's highest restrictions imposed in Saudi Arabia including the closure of recreational centers. During the COVID-19 curfew restrictions, the public should be encouraged to shift in outdoor physical activities to different intensities of home-based exercises. For instance, the nationwide Fit from Home (FFH) campaign, which was promoted by public health officials in Thailand during the lockdown period, illustrated improvement of desirable physical activities in Thai populations [30].

We would like to highlight the strengths of the present study. The present study used a valid and reliable usual dietary assessment tool for Saudis [20]. The physical activity questions used in the present study were based on the BRFSS Questionnaire [22]. In addition, the present study measured changes in both moderate and vigorous physical activity behaviors in Saudi adults during the COVID-19 curfew restrictions. Moreover, the effects of COVID-19 restrictions on the BMI, diet, and physical activity were assessed in a sample that represented Saudi adults from all regions, so the results of the study could be generalized to all the Saudi adult population.

However, our study has certain limitations. First, the demographic and anthropometric parameters of the study participants were self-reported in online questionnaires instead of measuring them personally. These could lead to decreased reliability and biasing on the part of the respondent. Second, the present study did not consider the psychological determinants in the design of the questionnaire, which may intervene in the correlation between behavior imposed by the curfew restrictions. In addition, the study questionnaire was relatively long, as the section of assessing dietary behavior changes included 208 items. Lastly, lifestyle changes may contribute to reasons besides the pandemic, so research similar to the present study performed in areas with minimal effect of the COVID-19 pandemic could give comparative results for a more valid conclusion.

## Conclusions

The present research findings showed that curfew restrictions probably proposed a chance for the communities to be at home and opt for healthy meal choices. Nevertheless, along with the extended duration of limited societal interactions, the deteriorating effect on the maintenance of regular physical activities could prove a remarkable public health consequence. Community healthcare providers are therefore guided to observe and evaluate physical well-being on population-based criteria. Especially, modified interceding to encourage healthy behaviors seems mandatory to promote the health of the communities afflicted by the pandemic.

## Appendices

Research questionnaire

Please fill in your response to the following.

Section 1: Demographic Data

1. Gender           Male                                Female             

2. What is your age?  ______________  years

3. What is your height? _________ centimeters

4. What is your current weight? _________ kilograms

5. What was your weight before the quarantine? _________ kilograms

6. What is your occupation?

    Student                         Employed                             Unemployed  

7. What is your current marital status?

    Single                      Married                         Divorced                             Widowed

8. What is the highest level of education you have completed? 

    Less than high school              High school or equivalent           

    Bachelor's degree                    Postgraduate degree          

9. What region are you from?

    Central region                 Western region                 Eastern region                                 

    Southern region              Northern region                Not applicable/outside Saudi Arabia                          

10. What is your current household income?

     Under SR 6,000                 SR 6,000-9,999               SR 10,000-13,999                                                

     SR 14,000-19,000              Over SR 19,000                                 

11. Has your household income changed during the quarantine?

     No                      Yes: increased income                      Yes: decreased income

Section 2: Food Frequency Consumption

**Table 6:** Food frequency consumption

Average food consumed prior to and during the quarantine											Food item
6+ daily	4-5 daily		2-3 daily	Once a day	5-6 per week	2-4 every week	Once a week	1-3 times per month	Never taken or less than once a month		
											Meat and fish
										Prior to the quarantine	Lamb (kabsa)
										During the quarantine	
										Prior to the quarantine	Grilled meat sautés
										During the quarantine	
										Prior to the quarantine	Meat kebab
										During the quarantine	
										Prior to the quarantine	Edam meat (dish)
										During the quarantine	
										Prior to the quarantine	Chicken (kabsa)
										During the quarantine	
										Prior to the quarantine	Chicken (fried) (e.g., Proust)
										During the quarantine	
										Prior to the quarantine	Chicken (grilled)
										During the quarantine	
										Prior to the quarantine	Chicken kebab
										During the quarantine	
										Prior to the quarantine	Edam chicken (dish)
										During the quarantine	
										Prior to the quarantine	Camel meat (Hashi)
										During the quarantine	
										Prior to the quarantine	Beef
										During the quarantine	
										Prior to the quarantine	Sausage
										During the quarantine	
										Prior to the quarantine	Shawarma (dish)
										During the quarantine	
										Prior to the quarantine	Kebdah (dish)
										During the quarantine	
										Prior to the quarantine	Fried fish
										During the quarantine	
										Prior to the quarantine	Grilled fish
										During the quarantine	
										Prior to the quarantine	Canned fish (tuna)
										During the quarantine	
										Prior to the quarantine	Shrimp
										During the quarantine	
											Bread and cereals
										Prior to the quarantine	White rice
										During the quarantine	
										Prior to the quarantine	Biryani rice or red
										During the quarantine	
										Prior to the quarantine	White bread
										During the quarantine	
										Prior to the quarantine	Brown bread
										During the quarantine	
										Prior to the quarantine	Bread with cereal
										During the quarantine	
										Prior to the quarantine	Pies (cheese pie)
										During the quarantine	
										Prior to the quarantine	Pizza (one piece)
										During the quarantine	
										Prior to the quarantine	Pasta
										During the quarantine	
										Prior to the quarantine	Breakfast cereals (corn flakes)
										During the quarantine	
										Prior to the quarantine	Pastries (croissant)
										During the quarantine	
										Prior to the quarantine	Mashed potatoes
										During the quarantine	
										Prior to the quarantine	Boiled potatoes
										During the quarantine	
										Prior to the quarantine	French fries
										During the quarantine	
										Prior to the quarantine	Easidah (porridge)
										During the quarantine	
										Prior to the quarantine	Maesub
										During the quarantine	
											Sandwiches and burgers
										Prior to the quarantine	Egg sandwich (fried)
										During the quarantine	
										Prior to the quarantine	Egg sandwich (boiled)
										During the quarantine	
										Prior to the quarantine	Kebdah sandwich
										During the quarantine	
										Prior to the quarantine	Meat sandwich
										During the quarantine	
										Prior to the quarantine	Chicken sandwich
										During the quarantine	
										Prior to the quarantine	Falafel sandwich
										During the quarantine	
										Prior to the quarantine	Meat burger
										During the quarantine	
										Prior to the quarantine	Chicken burger
										During the quarantine	
										Prior to the quarantine	Shawarma
										During the quarantine	
											Dairy and fat
										Prior to the quarantine	Liquid cheese
										During the quarantine	
										Prior to the quarantine	White cheese
										During the quarantine	
										Prior to the quarantine	Low-fat cheese
										During the quarantine	
										Prior to the quarantine	Full-fat yogurt
										During the quarantine	
										Prior to the quarantine	Low-fat yogurt
										During the quarantine	
										Prior to the quarantine	Skimmed yogurt
										During the quarantine	
										Prior to the quarantine	Full-fat cream
										During the quarantine	
										Prior to the quarantine	Low-fat cream
										During the quarantine	
										Prior to the quarantine	Skimming cream
										During the quarantine	
										Prior to the quarantine	Full-fat labneh
										During the quarantine	
										Prior to the quarantine	Low-fat labneh
										During the quarantine	
										Prior to the quarantine	Fat-free labneh
										During the quarantine	
										Prior to the quarantine	Full-fat milk (cup)
										During the quarantine	
										Prior to the quarantine	Low-fat milk (cup)
										During the quarantine	
										Prior to the quarantine	Skimmed milk (cup)
										During the quarantine	
										Prior to the quarantine	Full-fat milk (cup)
										During the quarantine	
										Prior to the quarantine	Low-fat milk (cup)
										During the quarantine	
										Prior to the quarantine	Skimmed milk (cup)
										During the quarantine	
										Prior to the quarantine	Cream caramel
										During the quarantine	
										Prior to the quarantine	Eggs (boiled)
										During the quarantine	
										Prior to the quarantine	Eggs (fried)
										During the quarantine	
										Prior to the quarantine	Mayonnaise (1 tablespoon)
										During the quarantine	
										Prior to the quarantine	Butter (teaspoon)
										During the quarantine	
										Prior to the quarantine	Margarine (teaspoon)
										During the quarantine	
											Desserts and snacks
										Prior to the quarantine	Ice cream
										During the quarantine	
										Prior to the quarantine	Chocolate bars
										During the quarantine	
										Prior to the quarantine	Potato chips
										During the quarantine	
										Prior to the quarantine	Nuts
										During the quarantine	
										Prior to the quarantine	Salted biscuits
										During the quarantine	
										Prior to the quarantine	Sweet biscuits
										During the quarantine	
										Prior to the quarantine	Light fruit cakes
										During the quarantine	
										Prior to the quarantine	Cakes and pastries (doughnuts)
										During the quarantine	
										Prior to the quarantine	Sweets (e.g., gum and toffee)
										During the quarantine	
										Prior to the quarantine	Chocolate sweets
										During the quarantine	
										Prior to the quarantine	Mahalbiya (rice pudding)
										During the quarantine	
										Prior to the quarantine	Sugar added to drinks (small spoon)
										During the quarantine	
										Prior to the quarantine	Vegetable soup (bowl)
										During the quarantine	
										Prior to the quarantine	Hummus (dish)
										During the quarantine	
										Prior to the quarantine	Tomato paste (ketchup)
										During the quarantine	
										Prior to the quarantine	Pickles (1 tablespoon)
										During the quarantine	
										Prior to the quarantine	Jam (teaspoon)
										During the quarantine	
										Prior to the quarantine	Honey (teaspoon)
										During the quarantine	
										Prior to the quarantine	Peanut butter (1 teaspoon)
										During the quarantine	
										Prior to the quarantine	Sauces (tablespoon) (ranch sauce and cheese sauce)
										During the quarantine	
											Drinks
										Prior to the quarantine	Red tea (cup)
										During the quarantine	
										Prior to the quarantine	Green tea (cup)
										During the quarantine	
										Prior to the quarantine	Arabic coffee (cup)
										During the quarantine	
										Prior to the quarantine	Instant coffee or cappuccino (cup)
										During the quarantine	
										Prior to the quarantine	Caffeine-free coffee (cup)
										During the quarantine	
										Prior to the quarantine	Cocoa or hot chocolate (cup)
										During the quarantine	
										Prior to the quarantine	Soft drink (can)
										During the quarantine	
										Prior to the quarantine	Low-calorie soft drink (can)
										During the quarantine	
										Prior to the quarantine	100% fruit juice (cup)
										During the quarantine	
										Prior to the quarantine	Fruit syrup (can)
										During the quarantine	
										Prior to the quarantine	Fruit flavor syrup (cup)
										During the quarantine	
											Fruits
										Prior to the quarantine	Dates (3 fruits)
										During the quarantine	
										Prior to the quarantine	Apple (1 fruit)
										During the quarantine	
										Prior to the quarantine	Pear (1 fruit)
										During the quarantine	
										Prior to the quarantine	Mango (1 fruit)
										During the quarantine	
										Prior to the quarantine	Grape (1 cluster)
										During the quarantine	
										Prior to the quarantine	Orange or tangerine (1 fruit)
										During the quarantine	
										Prior to the quarantine	Pineapple (1 slice)
										During the quarantine	
										Prior to the quarantine	Melon (1 slice)
										During the quarantine	
										Prior to the quarantine	Banana (1 fruit)
										During the quarantine	
										Prior to the quarantine	Watermelon (slice)
										During the quarantine	
										Prior to the quarantine	Peach, plum, and apricot (1 fruit)
										During the quarantine	
										Prior to the quarantine	Strawberries (1 cup)
										During the quarantine	
										Prior to the quarantine	Berries (1 cup)
										During the quarantine	
										Prior to the quarantine	Avocado (1 fruit)
										During the quarantine	
										Prior to the quarantine	Kiwi (1 fruit)
										During the quarantine	
										Prior to the quarantine	Canned fruits (peaches and pineapples) (1 cup)
										During the quarantine	
										Prior to the quarantine	Dried fruits (raisins) (1 cup)
										During the quarantine	
											Vegetables
										Prior to the quarantine	Green salad (1 cup)
										During the quarantine	
										Prior to the quarantine	Cucumber (1 cup)
										During the quarantine	
										Prior to the quarantine	Pumpkin (1 cup)
										During the quarantine	
										Prior to the quarantine	Spinach (1 cup)
										During the quarantine	
										Prior to the quarantine	Broccoli (1 cup)
										During the quarantine	
										Prior to the quarantine	Cabbage (1 cup)
										During the quarantine	
										Prior to the quarantine	Peas (1 cup)
										During the quarantine	
										Prior to the quarantine	Beans (1 cup)
										During the quarantine	
										Prior to the quarantine	Squash (1 cup)
										During the quarantine	
										Prior to the quarantine	Cauliflower (1 cup)
										During the quarantine	
										Prior to the quarantine	Leeks (1 cup)
										During the quarantine	
										Prior to the quarantine	Onion (1 cup)
										During the quarantine	
										Prior to the quarantine	Garlic (1 cup)
										During the quarantine	
										Prior to the quarantine	Mushroom (1 cup)
										During the quarantine	
										Prior to the quarantine	Peppers (1 cup)
										During the quarantine	
										Prior to the quarantine	Carrot (1 cup)
										During the quarantine	
										Prior to the quarantine	Lettuce (1 cup)
										During the quarantine	
										Prior to the quarantine	Tomato (1 cup)
										During the quarantine	
										Prior to the quarantine	Corn (1 cup)
										During the quarantine	
										Prior to the quarantine	Beetroot (1 cup)
										During the quarantine	
										Prior to the quarantine	Lentil (1 cup)
										During the quarantine	

Section 3: Physical Activity

If you have a job, please answer questions 1-7. If you do not have a job, skip question 1 and answer questions 2-7.

1. When you are at work, which of the following best describes what you do?

If you have multiple jobs, include all jobs.

   Mostly sitting or standing              Mostly walking     

   Mostly heavy labor or physically demanding work                   

For questions 2-7, we are interested in two types of physical activity: moderate and vigorous. Moderate activities cause small increases in breathing or heart rate, while vigorous activities cause large increases in breathing or heart rate.

2. Now, thinking about the moderate activities you do when you are not working in a usual week, do you do moderate activities for at least 10 minutes at a time, such as brisk walking, bicycling, vacuuming, gardening, or anything else that causes some increase in breathing or heart rate?

   No               Yes (Go to question 5)          

3. How many days per week do you do these moderate activities for at least 10 minutes at a time?

_________ days per week

    Do not do any moderate physical activity for at least 10 minutes at a time (Go to question 5)

4. On days when you do moderate activities for at least 10 minutes at a time, how much total time per day do you spend doing these activities?  

________ minutes per day                                 

5. Now, thinking about the vigorous activities you do when you are not working in a usual week, do you do vigorous activities for at least 10 minutes at a time, such as running, aerobics, heavy yard work, or anything else that causes large increases in breathing or heart rate?

    No                    Yes                

6. How many days per week do you do these vigorous activities for at least 10 minutes at a time?

________ days per week

    Do not do any vigorous physical activity for at least 10 minutes at a time

7. On days when you do vigorous activities for at least 10 minutes at a time, how much total time per day do you spend doing these activities?  

________ minutes per day            
